# Dynamic Cross Talk between S1P and CXCL12 Regulates Hematopoietic Stem Cells Migration, Development and Bone Remodeling

**DOI:** 10.3390/ph6091145

**Published:** 2013-09-23

**Authors:** Karin Golan, Orit Kollet, Tsvee Lapidot

**Affiliations:** Department of Immunology, Weizmann Institute of Science, 234 Herzl Str., Rehovot 76100, Israel; E-Mails: karin.golan@weizmann.ac.il (K.G.); orit.kollet@weizmann.ac.il (O.K.)

**Keywords:** hematopoietic stem cells, CXCL12/CXCR4, S1P, mobilization, bone remodeling

## Abstract

Hematopoietic stem cells (HSCs) are mostly retained in a quiescent non-motile mode in their bone marrow (BM) niches, shifting to a migratory cycling and differentiating state to replenish the blood with mature leukocytes on demand. The balance between the major chemo-attractants CXCL12, predominantly in the BM, and S1P, mainly in the blood, dynamically regulates HSC recruitment to the circulation versus their retention in the BM. During alarm situations, stress-signals induce a decrease in CXCL12 levels in the BM, while S1P levels are rapidly and transiently increased in the circulation, thus favoring mobilization of stem cells as part of host defense and repair mechanisms. Myeloid cytokines, including G-CSF, up-regulate S1P signaling in the BM via the PI3K pathway. Induced CXCL12 secretion from stromal cells via reactive oxygen species (ROS) generation and increased S1P_1_ expression and ROS signaling in HSCs, all facilitate mobilization. Bone turnover is also modulated by both CXCL12 and S1P, regulating the dynamic BM stromal microenvironment, osteoclasts and stem cell niches which all functionally express CXCL12 and S1P receptors. Overall, CXCL12 and S1P levels in the BM and circulation are synchronized to mutually control HSC motility, leukocyte production and osteoclast/osteoblast bone turnover during homeostasis and stress situations.

## 1. Introduction

Blood forming stem cells reside mainly in the BM, replenishing our body with new blood and immune cells on demand. Most stem cells are retained quiescent in a non-motile mode, anchored to specialized BM stromal niches. This microenvironment prevents their motility and differentiation via adhesive interactions. These interactions are dynamic and must be altered in order for stem and progenitor cells to proliferate and differentiate, while maintaining a constant pool of primitive cells throughout adult life. Stem cell proliferation and differentiation in the BM is accompanied by immature and maturing leukocyte egress to the circulation. This process is dramatically accelerated during alarm situations induced by injury, bleeding, viral or bacterial inflammations and DNA damage, as part of host defense and repair mechanism [1,2,3,4,5,6,7]. Stress-induced recruitment is mimicked in clinical stem cell mobilization protocols in order to induce proliferation and recruitment of progenitor cells to the circulation, from which they are harvested for BM transplantation.

A variety of agents can induce stem cell mobilization, such as myeloid cytokine granulocyte colony stimulating factor (G-CSF) [8,9,10], CXCR4 antagonist AMD3100 [11,12], sulfated polysaccharides [13,14] and chemotherapy drugs such as cyclophosphamide (Cy) and paclitaxel [3]. While G-CSF is repeatedly administrated for a few consecutive days, AMD3100 is a rapid mobilization agent that is given only once, yet both induce stem cell recruitment to the circulation by increasing their motility and reducing their retention in the BM microenvironment [15]. These dynamic changes during mobilization are achieved through a complex interplay between the immune and the nervous systems, the bone remodeling system (osteoblasts and osteoclasts) and the BM microenvironment. Homeostatic circadian rhythms, controlled by cycles of light and darkness via norepinephrine from the brain and nervous system, regulate not only stem cell migration from the BM to the circulation, but also bone remodeling processes [16]. Stress conditions involving signals from the nervous and immune systems also affect the activities of various bone cells and modify the repertoire of molecules implicated in blood forming stem and progenitor cells regulation [17].

The chemokine CXCL12 (also termed stromal cell derived factor 1, SDF-1) is constitutively expressed by murine and human BM stromal cells, such as perivascular, endothelial and endosteal bone lining stromal cells in the stem cell niche and recently was shown to be expressed also by osteoclasts [18]. CXCL12 is also a survival factor for stem and progenitor cells, and has been implicated in regulation of their homing, retention, development and recruitment to the circulation [19,20]. Under homeostatic conditions, CXCL12 concentration is relatively high in the BM, maintaining stem cells in a non-motile quiescent state, attached to BM stromal supporting cells. This is facilitated via activation of adhesion molecules, such as the integrins VLA-4/5 and LFA-1 [21] and the receptor CD44 and its major ligand hyaluronan [22]. The major receptor of CXCL12 is CXCR4, a G-protein-coupled receptor (GPCR) that is expressed on many cell types, including BM stem cells and stromal cells [23].

Sphingosine-1-phosphate (S1P) is a bioactive sphingolipid metabolite that has been implicated in cell migration, survival, proliferation and angiogenesis, as well as in immune responses [24]. It is generated from sphingosine by the action of sphingosine kinases (Sphks), and can either be converted back to sphingosine by specific S1P phosphatases (SPPs) or degraded by S1P lyase (SPL) to form phosphoethanolamine and hexadecenal [25]. Although most cells can synthesize S1P, its levels are maintained in the micromolar range in the blood and lymph circulations as compared to only nanomolar in solid tissues [26,27]. This is due to its degradation by the enzyme S1P lyase that is found at high levels in solid tissues but not in blood or lymph [28]. Concomitantly, S1P is highly produced by mature red blood cells [25,29,30], activated platelets [31] as well as by endothelial cells, which were recently discovered to be a major source for plasma S1P [32,33,34]. S1P is the ligand for a family of five specific seven transmembrane-spanning G-protein-coupled receptors (GPCRs), called S1P_1–5_. These S1P receptors couple to a variety of G proteins, enabling them to regulate numerous downstream signaling pathways including ERK and PI3K [35]. The functional response of different cells to S1P varies depending on their S1P receptor repertoire [24,25,36]. All S1P receptors are expressed on BM stem cells [37], as well as on BM stromal cells [38]. S1P is produced inside cells and therefore must be secreted to exert its effects through these receptors. Recent evidence suggests that S1P release is mediated by ATP binding cassette (ABC) transporters, like ABCC1 [25,39], from different cells and by spinster 2 (Spns2), a member of the major facilitator superfamily of non-ATP-dependent transporters, from endothelial cells. The plasma S1P concentration of SPNS2-deficient mice was reduced to approximately 60% as compared to WT mice and these mice were lymphopenic. Spns2 transporter was shown to play a role in regulation of plasma as well as lymph S1P levels and consequently to influences lymphocyte trafficking [32,33,40].

In this review, we will focus on the dynamic regulation of blood forming stem and progenitor cells as well as mature leukocyte motility by two major chemoattractants, CXCL12 and S1P, which are both regulated by Specificity Protein 1 (SP1) [16,41]. The tight balance between CXCL12 in the BM and S1P in the circulation, dictates the direction of stem cell movement. Nevertheless, the levels of these chemo-attractants in the body dramatically change upon stress-induced alarm situations to ensure stem cell mobilization as part of host defense and repair mechanisms. We will discuss the mutual, overlapping regulation of stem cell egress and mobilization by CXCL12 and S1P, as well as bone remodeling processes.

## 2. Migration of Hematopoietic Stem Cells and Mature Leukocytes is Dynamically Regulated by the Levels of CXCL12 in the BM and S1P in the Blood

Motility is a key feature of blood forming stem cells, that is orchestrated by various cytokines, chemokines, proteolytic enzymes and adhesion molecules [3,42,43]. This occurs through a dynamic interplay between the immune system and the bone microenvironment [4,16,17,44,45]. CXCL12 and its major receptor, CXCR4, are both essential for seeding of the BM by hematopoietic progenitors during embriogenesis and currently CXCL12 is considered as the most powerful chemoattractant of both human and murine stem cells [46,47,48]. Blood forming stem cell quiescence, maintenance and retention in the BM are dependent on CXCL12 levels [2,23,48,49,50] and global conditional deletion of this chemokine or its major receptor CXCR4 depletes stem cells from the BM [23,49]. Interestingly, CXCL12 via interactions with heparan sulfate proteoglycans can remain membrane-bound on the cell surface and extracellular matrix to facilitate homing as well as signaling. Mice with a genetic defect in such interactions, suffer from homeostatic and tissue repair problems [51], suggesting an important role for cell surface CXCL12. High BM CXCL12 levels maintain stem cells in a non-motile quiescent state via adhesive interactions with the stromal microenvironment. These interactions need to be broken in order for the cells to detach, enter the cell cycle and gain motility. Once stem cells are no longer bound to the microenvironment, they undergo proliferation and differentiation. Low BM CXCL12 levels will cause detachment of stem cells from their niche and release to the circulation, a process termed “egress”. In contrast, high BM levels of CXCL12 recruit endogenous or transplanted circulating stem cells back to the BM, as part of a multistep process named “homing”. CXCL12 also promotes the proliferation and maintenance of B-lineage progenitors [48,52] and common lymphoid progenitors (CLPs) [53]. In addition to being expressed by hematopoietic cells, CXCR4 is also functionally expressed by BM stromal cells, including endothelium, reticular adventitial cells, endosteal bone-lining osteoblasts and mesenchymal stem and progenitor cells [1,23,54,55]. This allows CXCL12 to regulate both the hematopoietic and stromal compartments. The level of CXCL12 in the BM is tightly regulated by many factors, such as hormones, cytokines, the nerve system and bone remodeling, as well as by cellular connectivity. Recently, it was shown that CXCL12 levels in the BM are subjected to circadian rhythms, thus changing over a period of 24 h and controlling stem cell steady state egress to the blood [16]. Interestingly, connexin gap-junctions between BM stromal cells form a dynamic syncytium that regulates CXCL12 secretion and cell surface expression via calcium transmission [56]. In addition, CXCR4 expressing BM endothelial cells can efficiently internalize circulating CXCL12 and translocate it to the BM, increasing stem cell homing [54]. Recently, a second receptor for CXCL12 was discovered, CXCR7 (also termed RDC1), that is expressed in embryonic neuronal and heart tissue, in hematopoietic cells and activated endothelium. However, CXCR7 acts as a specific scavenger for CXCL12, causing its internalization and degradation without inducing signaling [57].

The bioactive lipid sphingosine 1-phosphate (S1P) has emerged in the last decade as a novel chemoattractant for blood forming stem cells and mature leukocytes, although with a lower potency compared to CXCL12. Treatment with FTY720, an agonist of S1P receptors (except for S1P_2_) that causes their desensitization, internalization and degradation [58,59,60], induces immunosuppression by depleting lymphocytes from blood and lymph circulation. Interestingly, FTY720 was derived from an immunosuppressive natural product, myriocin (ISP-I), which is a fungus with properties of eternal youth in traditional Chinese medicine [61]. Subsequently, it was demonstrated that S1P is the major regulator for lymphocyte egress from the thymus and secondary lymphoid organs into circulatory fluids via one of its major receptors, S1P_1_, shown to be involved in cell motility and expressed in many cell types [62]. Interestingly, the S1P/S1P_1_ axis also contributes to B cell egress from the BM. Mice conditionally deficient in S1P_1_ in B lymphocytes exhibit accumulation of immature B cells in the BM parenchyma and a reduction of these cells in sinusoids and peripheral blood, suggesting reduced egress efficiency [63]. Blood forming stem cells express all S1P receptors on their cell surface, although their pattern and levels of expression differ between primitive and more committed progenitors. For example, S1P_3_ and S1P_5_ are expressed predominantly on immature progenitors, while S1P_2_ and S1P_4_ are found mainly on committed progenitors [37]. Interestingly, S1P_1_, that is known to play a role in cell migration, is expressed highly on both immature and committed progenitors, suggesting its involvement in stem cell and leukocyte egress from the BM [37]. S1P can act directly as a chemoattractant for blood forming stem cells in a dose-dependent manner [64,65]. Importantly, stem cell chemotactic activity of the plasma was almost completely abolished upon inactivation of bioactive lipids present in the plasma, suggesting a crucial role for S1P in the circulation as a chemo-attractant for BM-residing stem cells [65]. Desensitization of S1P receptors by FTY720 reduced stem cell steady state egress in mice [38,66,67]. In accordance, interference with the gradient of S1P between the blood and different tissues by 4-deoxypyridoxine (DOP), which inhibits the activity of the degrading enzyme S1P lyase and therefore increases S1P concentrations in the BM but not in the blood [63], also inhibits homeostatic egress of stem cells [38]. Once blood forming stem cells enter the blood, they can either home back to the BM or follow extramedullary traffic routes [68]. As part of lymphatic traveling, stem cells traffic to multiple non-lymphatic peripheral organs where they might generate innate immune effector cells on demand. These migratory stem cells can reenter circulatory fluids via S1P_1_-dependent chemotaxis towards the high S1P concentration [67]. Thus, S1P is a major regulator of stem cell egress not only from the BM but also from extramedullary organs [69].

It was proposed that S1P receptors expressed on primitive hematopoietic cells can signal through the PI3K pathway, thereby activating Rho GTPases and Vav-1 as part of the regulation of primitive cell motility [70]. Recently, it was shown that Vav-1 deficient murine stem cells have an impaired response to CXCL12 and also reduced homing [71]. Based on these studies, we hypothesize that there is mutual regulation between S1P and CXCL12 with regards to stem cell motility. In accordance, some of the molecular mechanistic pathways that are activated by S1P receptors are also activated by CXCR4-induced CXCL12 signaling, suggesting a synergistic effect on cell motility. S1P together with CXCL12 were indeed reported to have a synergistic effect on the migration of hematopoietic progenitor cells [70], however, this finding was later refuted by a different group showing no additive effect when both chemoattractants are added to the same plate [72]. In contrast, this group showed that over-expression of S1P_1_ on immature human CD34^+^ cells strongly reduces their migration towards a gradient of CXCL12 and *in vivo* homing via inhibition of CXCR4 signaling. We suggest that in a physiologic environment, S1P and CXCL12 may also have synergistic effects, which are driven by co-localization of CXCR4 and some of S1P receptors in lipid rafts, thus allowing both chemo-attractants to bind to their receptors and induce a stronger effect.

Recent studies show a major role for the sympathetic nervous system in stem cell regulation of migration, as well as development [73,74]. It was shown that the sympathetic nervous system can directly stimulate human HSPCs motility and proliferation [45] in addition to its indirect effect on the murine stroma microenvironment [75,76]. The levels of CXCL12 in the BM are regulated via light and dark cues through the sympathetic nervous system. As such, circadian rhythms of CXCL12 dictate the steady state egress of stem cells from the BM to the circulation. The peak in the number of circulating murine stem cells occurs early in the morning, when CXCL12 is low in the BM and the nadir at night, when BM CXCL12 is high [16,77]. This regulation by the nervous system is mediated through SP1, a circadian expressed transcription factor of CXCL12. Interestingly, SP1 is also the transcription factor of sphingosine kinase 1 (Sphk1), a biosynthetic enzyme of S1P [41]. Our preliminary data suggest that S1P in the circulation is also regulated in a circadian manner to further direct the homeostatic egress of stem cells. However, this topic is currently under investigation and future studies will reveal whether S1P has a role in circadian HSPC egress. Circadian regulation by the nervous system contributes also to bone turnover, which indirectly modulates stem cell motility and development [78].

**Figure 1:**
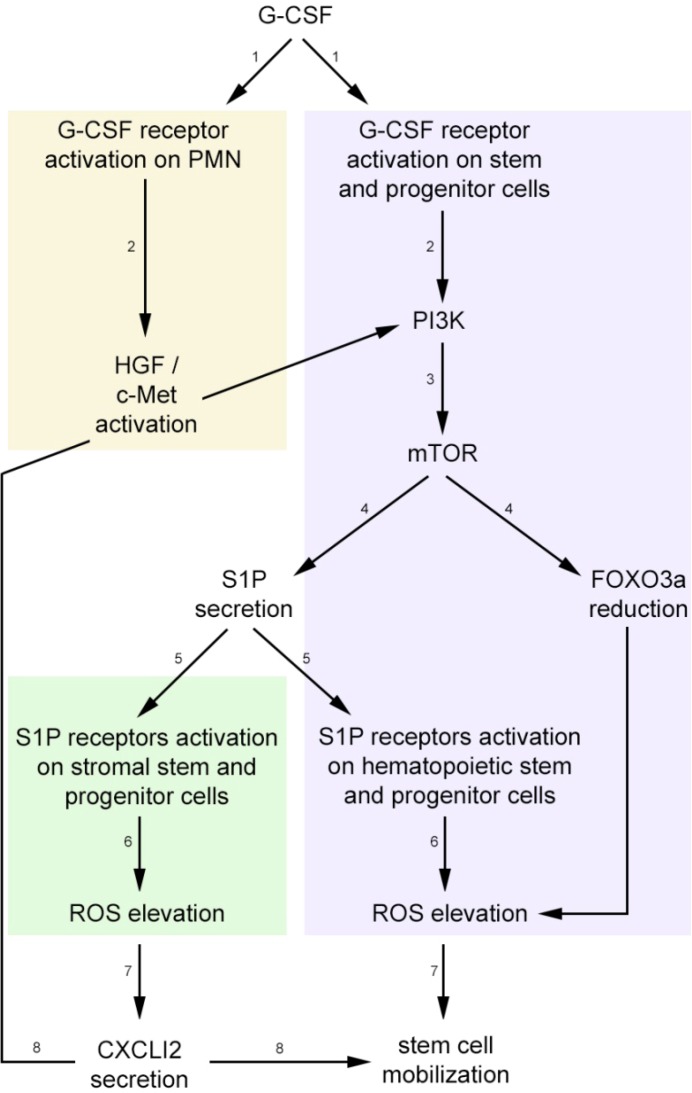
Flow chart of CXCL12 and S1P regulation during G-CSF-induced mobilization of stem cells. Upon G-CSF administration, it activates its receptors on stem cells and polymorphonuclear cells (PMN), activating HGF/c-Met. Such activation induces PI3K signaling via mTOR and FOXO3a reduction, leading to S1P production and secretion from BM cells [38]. S1P in turn can bind to its receptors both on stem cells thus leading to ROS generation and also on BM stromal progenitor cells to further facilitate CXCL12 secretion. CXCL12 can activate PI3K via HGF/c-Met signaling to further facilitate stem cell mobilization. The numbers in this suggested model represent the sequence of events following G-CSF administration in PMN cells, HSPCs and stromal stem and progenitor cells.

All together, blood forming stem cell motility is directed by both CXCL12 and S1P levels and the balance between these two important chemoattractants directs cell motility to the required location. As such, high BM CXCL12 levels will induce homing of stem cells and adhesion in their niche compartments, while increased S1P levels in the circulation and/or decreased CXCL12 levels in the BM will induce recruitment of stem cells to the circulation (Figure 1).

## 3. Stress-Induced Stem and Progenitor Cell Mobilization is Orchestrated by Dynamic CXCL12 and S1P Regulation via ROS Signaling

Blood forming stem and progenitor cells, as well as maturing leukocytes, pave their way from the BM reservoir to the circulation at high rates upon stress-induced alarm situations as a part of host defense and repair mechanisms [4,8,10,17]. Stem and progenitor cell mobilization can be clinically or experimentally induced by a variety of cytokines and chemokines [3,42]. Most commonly used is the myeloid cytokine G-CSF [8] and recently also the CXCR4 antagonist AMD3100 [79]. Mechanisms of G-CSF-induced mobilization consist of induction of proliferation and differentiation of quiescent stem cells, thus increasing the BM reservoir, accompanied by a decrease in stem cell retention in their BMmicroenvironment [9]. Following G-CSF administration, CXCL12 levels in the BM are transiently increased, followed by their rapid degradation and decrease at both protein [2,80] and mRNA [81] level. Nevertheless, G-CSF-induced mobilization increases CXCR4 receptors on BM stem and progenitor cells via HIF1α production [82]. This allows them to bind the transiently increased levels of CXCL12, increasing their motility and cell cycling. These intensified SDF-1/CXCR4 interactions further facilitate stem cell differentiation and motility by enhanced production of reactive oxygen species (ROS) through activation of the HGF/c-Met pathway [83]. ROS are oxygen derivatives containing free radical molecules that are produced mainly by mitochondria during cellular respiration and is dramatically enhanced as part of inflammation [84]. During stress-induced mobilization, stem cell ROS levels are enhanced, inducing cell cycle progression and the cells proliferate and differentiate into short-term repopulating cells and further on to myeloid mature cells. Quiescent stem cells share low ROS levels, supporting their long-term repopulation ability [85,86]. Interestingly, ROS balanced levels are essential for normal hematopoiesis. Impaired homeostatic ROS levels in Akt1 and Akt2 deficient mice caused increased stem cell quiescence leading to failure of hematopoiesis [87]. Thus, balanced ROS levels are essential for stem cells to switch between a non-motile quiescent mode to an active migrating and proliferating/differentiating state, as part of homeostasis and stress-induced mobilization. In contrast to G-CSF, AMD3100 is a dynamic and transient rapid mobilizing agent that triggers *in vivo* CXCL12 secretion from BM CXCR4-expressing stromal cells, followed by its release from the BM to the circulation. This leads to uPA, MMP-9 and ROS activation as part of stem cell rapid mobilization [11,79,88,89].

Recently, we found that S1P signaling is essential for stem cell mobilization and that its levels are transiently elevated in the plasma and BM during AMD3100, as well as G-CSF-induced mobilization [38]. Since AMD3100 is a very rapid mobilization agent, we observed a transient elevation in S1P plasma levels within 5 to 15 min, restoring to basal levels after 1 hour at the peak of HSPC mobilization. Moreover, we also found transient augmented S1P plasma levels following treatment with G-CSF, confirming previous observations [65], reaching a peak at day 4 of treatment. Nevertheless, one report claims that there are no changes in S1P plasma levels after G-CSF-induced mobilization and we believe these differences are due to a different administration and measurement protocols [66]. While this group injected G-CSF for 4 consecutive days and measured S1P plasma levels 12 h after the last injection [66], we administrated G-CSF for 5 consecutive days and measured S1P levels 4 h after the last injection [38]. Due to the dynamic and transient nature of S1P, which we showed is triggered and elevated as early as 30 min after one G-CSF injection, we believe that an overnight delay between the G-CSF last injection enables the system to return S1P to normal levels. In addition, at the peak of G-CSF-induced stem cell mobilization, we detected increased S1P levels in the plasma, as well as in the BM by a similar fold, suggesting that the gradient towards the blood is maintained in steady state and perhaps is even augmented at earlier time points of G-CSF administration. Altogether, CXCL12 and S1P levels are dynamically regulated in the BM and circulation during stress-induced mobilization, preceding stem cell recruitment and facilitating the best conditions for this process.

Various molecular pathways were reported to play a role in the tight regulation of CXCL12 and S1P levels during stress-induced mobilization in the BM and circulation. Overlapping publications show that proteolytic enzymes are responsible for the overall decrease in BM CXCL12 levels, including during G-CSF administration, reaching lowest concentrations at the time of stem cell collection [2,90,91,92]. An alternative explanation is provided by the Link group [81], claiming that G-CSF through an indirect mechanism reduces osteoblast levels, resulting in decreased CXCL12 expression in the BM. The consequent attenuation of SDF-1/CXCR4 signaling ultimately leads to HSPCs mobilization. S1P elevated concentration in the BM and circulation was shown to be mTOR mediated. Previously reports showed that the PI3K/Akt pathway may be activated by S1P and its receptors [93,94]. Nevertheless, the PI3K/Akt molecular pathway was demonstrated to play an essential role in G-CSF-induced mobilization [95], through activation of HGF/c-Met and mTOR pathways, leading to ROS production [83]. Therefore, we suggest that S1P may contribute to stem cell mobilization and ROS generation during stress-induces situations via the PI3K/Akt molecular pathway. There are several sources for S1P enhanced production in the circulation, such as erythrocytes, platelets and endothelial cells [32,96,97]. Ratajczak and colleagues have shown that the complement cascade is activated in the BM during stress-induced stem cell mobilization mediated by several drugs, including G-CSF and AMD3100. During this process, certain bioactive cleavage fragments are released and a membrane attack complex is generated [98,99]. S1P elevation in the circulation may result from activation of the complement cascade and interactions of the membrane attack complex with erythrocytes, leading to their lysis [65]. Nevertheless, activation of the coagulation cascade during infections, injuries or G-CSF administration may also contribute to elevated S1P levels in the circulation, as it may be released from activated platelets [100,101]. Interestingly, S1P was recently shown to have a role in thrombopoiesis by regulating platelet secretion into the circulation [102,103]. Overall, different cells and molecular pathways are involved in the regulation of CXCL12 and S1P levels in the BM and circulation during stress-induced stem cell mobilization.

The dynamic gradients of CXCL12 and S1P between the blood and the BM are essential for stem cell recruitment. Inhibition of CXCL12/CXCR4 interactions during G-CSF or AMD3100 treatment reduced stem cell mobilization to the circulation [2,79]. S1P signaling attenuation either by FTY720 desensitization of its receptors or by DOP administration and attenuation of its gradient between the BM and the circulation, both decreased AMD3100-induced stem cell mobilization [27,38,65,66]. Recently, we showed an essential role for S1P and S1P_1_ in G-CSF-induced mobilization in addition to AMD3100. The levels of S1P_1_ are augmented upon G-CSF treatment and mice conditionally deficient for this receptor in the hematopoietic compartment, exhibit reduced G-CSF-induced mobilization [38]. However, the role of S1P during G-CSF administration is controversial. While we and others demonstrated that inhibition of S1P receptors during G-CSF administration decreased stem cell mobilization [38,65], others showed no effect for such an inhibition [66,67]. We believe that the controversy arises from the different protocols used for G-CSF administration as previously described for the fluctuations in S1P levels during G-CSF administration.

Most importantly, we found that S1P is an important regulator of ROS levels in hematopoietic stem cells, as well as BM stromal progenitor cells, to be described below in further details. ROS signaling is essential for stem cell mobilization during G-CSF administration and its inhibition during this process, strongly reduces stem cell recruitment to the blood [83]. Therefore, we claim that S1P is essential for G-CSF-induced mobilization, not only by inducing stem cell migration to the blood, but also by serving as an inducer of ROS signaling, further facilitating stem cell development and recruitment. We observed that S1P inhibition affects mainly the more primitive cells and less the committed progenitor cells. This may account for the discrepancy in results between the different research groups.

Many molecular pathways are involved in the process of stem cell recruitment by AMD3100 and G-CSF. Interestingly, CXCL12 and S1P signaling during stress-induced mobilization converge to induce both elevation and activation of ROS. We previously showed that CXCL12 secretion from BM-stromal cells induced HGF activation of the mTOR pathway, thus inhibiting FOXO3a expression and leading to increased ROS production during G-CSF-induced mobilization [83]. In addition, ROS signaling is also involved in AMD3100-induced mobilization and its inhibition led to reduced stem cell mobilization [79]. We found that S1P is an activator of ROS signaling and increased BM S1P levels during stress-induced stem cell mobilization are required for ROS activation in primitive SKL cells, thus leading to increased cell motility, as previously published [83]. Importantly, we demonstrated that ROS signaling has a regulatory role also in BM stem and progenitor stromal cells, which are part of the niche microenvironment that supports blood forming cell development, motility and self-renewal. *In vitro* S1P binding to its receptors expressed also on BM progenitor stromal cells, induces ROS activation, leading to CXCL12 secretion. Based on these findings, we assume that S1P further facilitates stem cell egress and mobilization via ROS-induced CXCL12 secretion. Interestingly, CXCL12 can induce PI3K pathway activation via HGF/c-Met, thus also leading to S1P secretion. Therefore, we suggest that CXCL12 may have a self-regulatory loop by further inducing S1P production that will cause CXCL12 secretion from BM stromal cells during stress-induced mobilization. Altogether, S1P and CXCL12 mutually regulate each other by intensifying their signaling and transiently regulating their levels to facilitate stem cell mobilization.

Stress-induced situations, such as mild bleeding and G-CSF or LPS treatments, trigger HSPCs mobilization concomitantly with a dynamic alteration of their BM microenvironment. For example, G-CSF or cyclophosphamide injections lead to disappearance or altered morphology of bone-lining osteoblasts, resulting in their reduced function [75,81,104,105] as well as to robust appearance of active osteoclasts in human and mice [18,75,92,106,107]. Bone formation and bone degradation, carried out by BM osteoblasts and monocyte-derived osteoclasts, respectively, are part of a complex process of bone remodeling. Since during stress-induced situations CXCL12 and S1P levels are also dynamically regulated, we suggest that the balance between these two factors contributes to the bone remodeling process that is coupled to HSPC egress and mobilization.

## 4. S1P and CXCL12 Regulate Bone Turnover and the Dynamic BM Microenvironment, Indirectly Facilitating Stem Cell Development and Migration

The major mammalian hematopoietic organ is the BM, a microenvironment harboring various stem cell niches that provide the regulatory milieu needed for stem cell long-term survival and function [108]. It is imperative to understand what is special in this organ that makes it the best “home” for long-term repopulating blood forming stem cells, and why bone disorders are often associated with pathologic conditions having hematopoietic implications. For example, osteoporosis in elderly individuals is associated with reduced host immunity. Although BM stem cell numbers are increased, their function is reduced. Considering elements available only in the BM for the unique life-span maintenance of stem cells, points at bone-maintaining cells, mainly osteoblasts and osteoclasts, and bone remodeling processes. Bone integrity and homeostasis are maintained throughout life via cycles of bone resorption and formation, coupled processes termed bone remodeling. Bone formation is carried out by osteoblasts, stromal cells that are the progeny of mesenchymal stem cells. Bone resorption is carried out by multinucleated osteoclasts, which are generated by fusion of monocytic precursors derived from hematopoietic stem cells [109]. Bone cell mutual communication, migration and differentiation are pivotal for orchestrating this multistep process of bone remodeling [110,111]. Osteoclast precursor differentiation and activation are promoted by osteoblasts and osteocytes [112] via secretion of the cytokines MCS-F and G-CSF and expression of RANKL [109,113]. These regulatory relationships are reciprocal, since osteoclasts regulate osteoblast differentiation and thus bone formation via signaling of Ephrin/Eph and semaphorins. These BM-located cross talks also influence stem cells [111]. The contribution of osteoblasts to stem cell support provided by the endosteal stem cell niche has been reported in various publications. An increase in osteoblast numbers correlated with expansion of the stem cell population, demonstrating involvement of osteoblastic PTH/PTHrP activated receptors and Jagged1/Notch1 [114], as well as bone morphogenic proteins, and N-cadherin/β-catenin signaling [115]. In line, specific ablation of early differentiating osteoblasts led to extramedular hematopoiesis, associated with reduced levels of lymphoid, erythroid and myeloid progenitors and moreover, stem cells in the BM. Interestingly, withdrawal of osteoblast-induced depletion, recruited hematopoiesis back to the medullary compartment [116]. Mesenchymal stem cells also contribute to the maintenance of the stem cell pool within the BM. We recently showed that *in vivo* treatment with bFGF increased the pool of BM stem cells via expansion of nestin-expressing stromal supportive cells, involving stem cell factor secretion, miR-31upregulation and CXCL12 reduction [117]. Osteoclasts also play a role in regulation of stem cells in the BM. We showed involvement of osteoclasts in the process of stem cell mobilization via CXCR4 and MMP-9, and also their activity in degrading essential niche-related components by cathepsin K [18]. Accordingly, we observed reduced stem and progenitor cell levels in the BM and increased extramedular localization of stem and progenitor cells in CD45 deficient mice, which have defective osteoclast fusion. This leads to mild osteopetrosis and abnormal structure of the trabecular bone, the region serving for stem cell lodgment [118]. Osteoclasts have been also implicated in mobilization of vascular progenitors by antagonizing the Wnt signaling pathway with DKK, which up-regulated RANKL expression within the BM [119]. However, Wnt signaling is essential for stem cell function, since DKK enforced expression reduced stem cell regenerative ability after transplantation [120].

S1P emerges as a principal signaling molecule in regulation of osteoblast-osteoclast communication and bone integrity. While the highest levels of S1P are recorded in the blood, the BM also contains certain amounts of S1P [38]. Both osteoclasts [121] and osteoblasts [122], produce S1P and functionally express its major receptors, S1P_1_ and S1P_2_ [123]. Moreover, both cell types respond to S1P stimulation. Osteoblast survival is facilitated by S1P, which prevents osteoblastic-cell apoptosis in a dose dependent manner [124]. Thus, secretion of S1P may be another regulation layer by which osteoclasts regulate osteoblast maturation and function. The ability of bone cells to produce and respond to S1P depends on their maturation stage. Following RANKL stimulation, differentiating osteoclasts increased expression of the S1P-catalizing enzyme Sphk1 [121,125] and subsequently, secretion of its product, active S1P. In accordance, Sphk1 inhibition, suppressed osteoclast differentiation. Interestingly, S1P integrates regulation of osteoclasts and osteoblasts, since it increases RANKL expression by osteoblasts and thus potentiates osteoclastogenesis [121]. Osteoclast-secreted S1P also serves to regulate bone formation. Specific deletion of the bone degrading enzyme CTK in murine osteoclasts resulted in mild osteopetrosis, a bone disease with unbalanced increased bone mass. Specifically, these CTK−/− osteoclasts exhibited up-regulated production of Sphk1, and S1P synthesis, which may explain the observed increase in osteoblasts [126], since S1P up-regulates osteoblast differentiation and bone formation [121]. In line, agonistic stimulation of S1P receptor in osteoblasts by S1P or FTY720 facilitated BMP-2-induced osteoblast differentiation of C2C12 myoblasts, via ERK1/2 and Smad1/5/8 phosphorylation [127].

Motility of osteoblasts and osteoclasts is also pivotal for their differentiation and function. Monocytic osteoclast precursors leave the BM, egress to the blood and migrate back following chemotactic signals of CXCL12 [128,129], which direct them to the endosteum, the stem cell-rich region connecting the bone and the marrow. In a murine model of multiple myeloma, CXCL12 secreted by the BM-invading multiple myeloma cells, recruited osteoclasts to the tumor vicinity and activated them, leading to bone loss [130]. Osteoblasts [1] and moreover MSC [23,131] express high levels of CXCL12 which contributes not only to stem cell survival but also to recruitment of osteoclast precursors to the bone surface [128,129]. Migration of these bone cells is also regulated by S1P, suggesting cross talk with CXCL12. Various monocytic cell subsets with osteoclastogenic potential, therefore considered as osteoclast precursors functionally express S1P_1_ and respond to an S1P gradient with positive chemotaxis. Thus, S1P chemoattracts *in vitro* osteoclast-precursor RAW264.7 murine cell line via S1P_1_ [132]. Interestingly, distinct S1P signaling via its two cognate receptors, S1P_1_ and S1P_2_ in osteoclasts was recently revealed. Osteoclast precursors also express S1P_2_, which mediates their negative chemotaxis or chemo repulsion from high S1P concentrations [133]. These high concentrations induced internalization of the positive chemoattraction mediator, S1P_1_. Hence, S1P dose directs receptor specific signaling, since S1P_1_ mediates positive chemotaxis in response to low S1P concentrations. S1P_2_ deficient mice suffer mild osteopetrosis and reduced bone resorption, demonstrating the role of this receptor in retaining osteoclasts within the BM. Indeed, antagonizing this receptor affected migration of monocytic cells including osteoclast precursors and cured osteoporosis by reducing bone-attachment of osteoclasts. These results demonstrate that bone remodeling is regulated by reciprocal activity of the S1P receptors, S1P_1_ and S1P_2_, via distinct Rac and Rho signaling, respectively [133]. Migration of human MSC and their osteogenic differentiation are also stimulated by S1P from osteoclast-conditioned medium [125]. This chemotactic effect is mediated by S1P_1_ and S1P_2_ via activation of JAK/STAT3 and FAK/PI3K/AKT signaling pathways, respectively. The expression of S1P_1_ and S1P_2_ was increased significantly when the MSC cells reached confluence [134]. Interestingly, we previously identified that CXCL12 secretion from BM MSC is upregulated when cells are in contact [56]. S1P regulates osteoclast adhesion as well. In vivo deletion of S1P_1_ in CD11b expressing monocytes and osteoclast precursors increased osteoclast adhesion to bones, exerting osteoporotic effects. In contrast, agonistic treatment with FTY720 reduced the number of bone-attached osteoclasts, relieving osteoporosis in mice [135]. Of interest, it is suggested that this reciprocal axis of activity regulates not only adhesive properties of osteoclast precursors to the bone, but also their entry and egress across the BM endothelial barrier [123,133].

Differentiation and motility of bone cells go hand in hand. For example, mature osteoblasts are recruited to sites of degraded bone in order to establish bone formation, guided by local cues, such as TGFβ [136] or PDGFbb [137] released from resorbed bone. However, pre-osteoblasts respond to S1P while mature osteoblasts do not. PDGF exerts positive chemotactic activity on pre-osteoblats, while S1P is a chemorepellent to these cells. Upon BMP2-mediated differentiation of osteoblast precursors, expression of S1P_2_ is repressed and the cells do not respond anymore to S1P but retain their positive chemotaxis to PDGF. Via regulation of S1P_2_ expression, S1P acts as a chemo-repellent, keeping mature osteoblasts within the BM to allow PDGF-guidance to bone fracture sites [137]. Thus, the balance between PDGF, S1P and osteoblast maturation state control osteoblast migration.

In summary, migration and development of stem cells and bone-remodeling cells share common regulators, particularly, S1P and CXCL12. Thus, location and function of these regulators integrate differentiation status and motility traits. These two systems are also guided by essential input from the nerve system via circadian rhythms, which regulate leukocyte migration and bone remodeling [138,139,140]. This mode of activity is inevitable due to the dynamic nature of this microenvironment, which is set to quickly adjust to extensive blood cell production, intensive maintenance of the calcium reservoir, remodeling of the skeleton and incidental immune challenges. Nevertheless, there is an overlap between many players contributing to both bone development and HSPC motility. Therefore, future studies will reveal a more complete and accurate mechanistic model for the mutual regulation of HSPC mobilization and bone remodeling during stress-induced situations, a topic which is currently controversial.

## 5. Conclusions, Clinical Aspects and Future Directions

**Figure 2:**
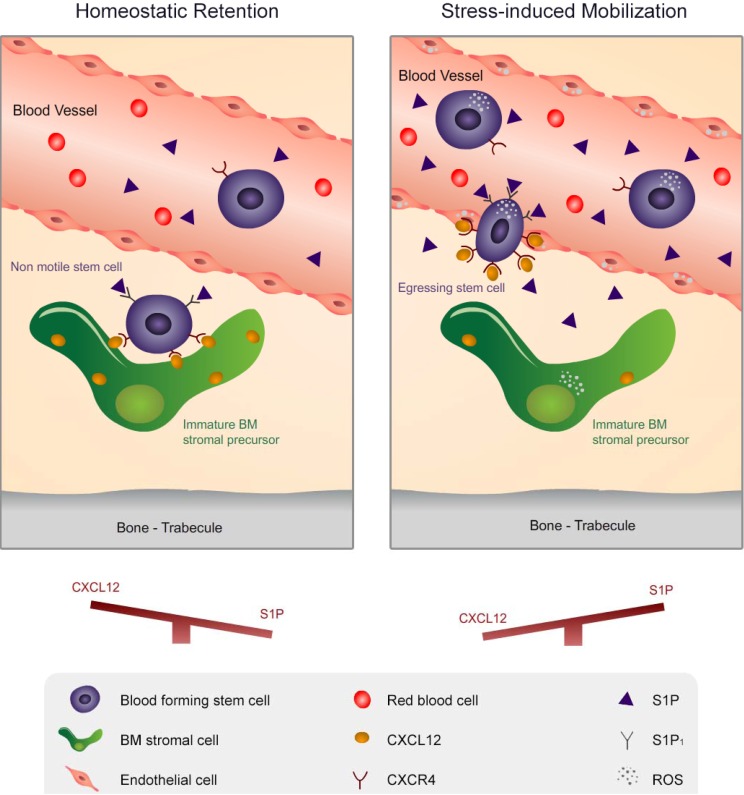
A schematic model for CXCL12 and S1P regulation during homeostatic retention and stress-induced mobilization. left panel: During steady state conditions, CXCL12 is highly expressed on BM stromal progenitor cells, maintaining stem cells in a non-motile quiescent state. There is a competition between plasma S1P and BM CXCL12 concentrations, determining the low stem cell egress levels. right panel: During stress-induced mobilization, CXCL12 levels are decreased in the BM, allowing detachment of stem cells from the BM stroma and switching to a motile proliferative/differentiating state. Concomitantly, S1P levels are increased in the BM and plasma, thus facilitating ROS signaling in stem cells to increase their active motile state as well as in BM stromal progenitor cells to further facilitate CXCL12 secretion.

BM transplantation procedures, which are routinely used to treat patients with hematological malignancies, as well as inherited genetic disorders, require mobilized stem cell collection from the circulation. Therefore, characterization of new regulators for stem cell development and mobilization has become a major focus in recent decades. For many years, CXCL12 was thought to be the only powerful chemo-attractant for stem cells, while more recently S1P has emerged as an additional chemo-attractant for these cells, although with a lower potency than CXCL12. The tightly regulated balance between these two chemo-attractants determines the location of stem cells and their state of motility and quiescence. During homeostasis, CXCL12 concentration is high in the BM, maintaining stem cells in a quiescent non-motile state. However, low numbers of blood forming stem cells egress to the circulation as part of physiological behavior. S1P levels are low in the BM and high in the circulation and therefore, exhibit a gradient that guides stem cells towards the circulation and allows them to escape from their non-motile state (Figure 2).

Overall, the balance between the levels of CXCL12 in the BM and S1P in the circulation dictates the steady state egress of stem cells. In contrast to homeostatic conditions where CXCL12 and S1P compete for stem cell retention in the BM versus egress to the circulation, during stress-induced conditions CXCL12 and S1P work hand in hand to facilitate stem cell mobilization as part of host defense and repair mechanisms. During such alarm situations, CXCL12 levels are overall decreased in the BM, while S1P concentration is increased in the BM as well as in the circulation, maintaining the strong gradient towards the circulation. These conditions are optimal for stem cells to exit their quiescent non-motile state and become recruited to the blood. Interestingly, there is a mutual tight regulation between CXCL12 and S1P, both aiming to elevate ROS levels in stem cells, thus inducing their mobilization and also in BM stromal cells to further facilitate CXCL12 secretion and reduction of stem cell retention to their niches (Figure 2). S1P has also an important role in bone remodeling, in terms of osteoblast and osteoclast differentiation and migration. By controlling the bone turnover, it indirectly regulates stem cell development and motility. Altogether, CXCL12 and S1P are master regulators that determine blood forming stem cells motility via chemoattraction, as well as via bone remodeling.

Since S1P is an essential regulator of mature leukocytes, its role in different autoimmune diseases, such as multiple sclerosis and rheumatoid arthritis, as well as cancer, has been determined over the last decades. FTY720 (fingolimod or Gilenya), a modulator of S1P receptors that inhibits S1P-dependent stem cell and lymphocyte egress, was first administrated as an immunosuppressive drug [141]. Later, it was found also to be a very efficient drug for multiple sclerosis, the most common inflammatory disorder of the central nervous system, by reducing trafficking of pathogenic inflammatory cells [142]. However, a major effect ascribed to FTY720 treatment is cardiovascular risk. FTY720 may cause decreased heart rate, bradycardia, after a single dose and may point to other longer-term risks [143]. Recently, S1P was suggested to play a role in rheumatoid arthritis (RA) as well [144]. Patients with RA share elevated levels of S1P in synovial fluids, as well as alterations in S1P signaling that leads to synovial fibroblast migration, proliferation, survival and production of pro-inflammatory cytokines/chemokines. FTY720 administration suppresses disease progression in arthritic mice by decreasing pro-inflammatory cytokines/chemokines secretion and the number of peripheral blood lymphocytes [144]. S1P is also involved in cancer progression, including cell transformation, survival, metastasis and tumor microenvironment neovascularization. Accumulating data show increased levels and activity of Sphingosine kinase 1, one of the biosynthetic enzymes of S1P, in cancers of the stomach, lung, brain, colon, kidney, breast and non-Hodgkin’s lymphoma. This enzyme behaves as an oncogene and appears to mediate the mitogenic effects of Ras [145]. Elevated S1P levels in cancers affect patient outcome and prognosis, tumor progression, and chemotherapy resistance. Interestingly, an important role for S1P was described in hematological malignancies, such as different types of leukemia, lymphoma and multiple myeloma [146]. Finally, manipulation of the S1P/S1P_1_ axis may be used to improve clinical mobilization protocols. As suggested by Bendall and colleagues, activation of S1P_1_ by the SEW2871 specific agonist during AMD3100 administration led to increased levels of mobilized stem cells that are harvested for BM transplantation [66].

Future studies focusing on the molecular aspects of S1P/S1P_1_ axis, leading to stem cell detachment from their BM stromal niches should be investigated. Understanding the complex and important cross talk between CXCL12 and S1P, in terms of mutual regulation, synergistic effects and molecular pathways involved in their regulation, will also enable a better understanding and manipulation of stem cells for therapeutic purposes. Most importantly, are there additional potent chemoattractants yet to be discovered for blood forming stem cells migration? What will happen if both CXCL12 and S1P are inhibited during stem cell-induced mobilization? Since CXCL12 was previously shown to be translocated by endothelial cells from the circulation to the BM, it will be interesting to test whether this is also true for S1P. If so, which are the receptors or transporters that facilitate this process? We recently established a new role for S1P in BM stromal progenitor cell regulation via activation of ROS signaling [38]. However, much is still to be learned regarding S1P regulation of the BM microenvironment, especially regarding the stem cell niche. Overall, both CXCL12 and S1P are essential regulators of stem cell development and migration, as well as of bone remodeling that share common signaling pathways and mutual regulatory loops, facilitating stem cell egress and stress-induced mobilization.
